# An Analysis of Risk Factors for Developing Hepatocellular Carcinoma in a Group of Hepatitis C Patients with Stage 3 Fibrosis following Interferon Therapy

**DOI:** 10.4137/cin.s644

**Published:** 2008-04-23

**Authors:** Sabina Mahmood, Kazumi Togawa, Miwa Kawanaka, Gouichi Niiyama, Gotaro Yamada

**Affiliations:** Department of Internal Medicine, Center for Liver Disease, Kawasaki Hospital, Kawasaki Medical School, Okayama 700-0986, Japan

**Keywords:** long prognosis, chronic hepatitis C, alanine aminotransferase, anti-inflammatory drugs, hepatocellular carcinoma

## Abstract

The risk of Hepatocellular carcinoma (HCC) is high in HCV-infected patients who have biochemically and histologically active chronic hepatitis. To observe the long prognosis of Chronic Hepatitis C (CHC) patients with stage 3 fibrosis (F3), 55 CHC patients after initial Interferon (IFN) therapy were followed up for up to 12 years (average 9.8 ± 2.3 years). According to the annual average alanine aminotransferase (ALT) levels, patients were grouped into, low (ALT ≦ 30 IU/l); moderate (ALT >30 <80 IU/l) and high (ALT ≧ 80 IU/l) ALT groups. Eleven patients were re-treated with IFN. During the follow-up period of 12 years, HCC developed in 26 patients with an average annual incidence of 3.9%. Biochemical responders to initial IFN therapy (n = 8) and those re-treated with IFN (n = 10), except 1, did not develop HCC. Cox regression analysis to evaluate risk factors for HCC occurrence, found development of Liver Cirrhosis within 3 years of initial IFN therapy(*P* = 0.05) and the 3 year annual average ALT post initial IFN therapy (*P* = 0.033) to be significant. The 12 year annual average ALT was also found to be significantly related to HCC occurrence (*P* = 0.016), on univariate analysis. Patients belonging to the continuously low ALT group (ALT ≦ 30 IU/l for ≧3 years), did not develop HCC or receive IFN re-treatment. In CHC patients with F3, after initial IFN therapy, keeping ALT continuously low, below 30 IU/l for 3 years or more seems important. Continuing treatment with anti-inflammatory drugs along with subsequent IFN re-treatment may prevent or delay HCC even in elderly patients.

## Background

Chronic infection with HCV (ongoing HCV infection for 6 months or more) is a leading cause of chronic liver disease, cirrhosis (LC) and hepatocellular carcinoma (HCC). In Japan the number of deaths annually due to HCC exceeds 30,000 (1). The risk of HCC development has been reported to be high in HCV-infected patients who have biochemically and histologically active chronic hepatitis, suggesting that necro-inflammation and associated regenerative processes play a pivotal role in hepatic carcinogenesis (2,3,4). Patients with advanced fibrosis (stage 3 or stage 4) are reported to be at a higher risk of development of HCC (5) and HCC patients have a significantly higher level of mean serum ALT than non-HCC patients (6). In long term follow-up studies, patients with persistently low or normal ALT develop HCC less frequently than those with persistently high ALT (5,7). Interferon (IFN) is an approved anti-viral agent known to be effective against HCV infection, by eliminating HCV and reducing ALT activity (8 ~ 13). However, only 50% of patients achieve sustained virological response (undetectable HCV RNA 6 months after the end of IFN treatment (14 ~ 17), the therapy regime extending from a minimum period of 4 weeks to a maximum period of 24 weeks. Immediate re-treatment with IFN or other anti-inflammatory drugs in patients who do not respond to initial IFN therapy, is sometimes effective. In our previous study (18) we followed 48 chronic hepatitis C (CHC) patients who were either partial responders PR (ALT between 30–80 IU/l); or non-responders: NR (ALT >80 IU/l, longer than 6 months) to initial IFN therapy, and were given anti-inflammatory drugs (19) such as Stronger Neo-Minophagen (SNMC), Ursodeoxy-cholic acid (UDCA) and Shosaioko-to, to subside inflammation and suppress ALT activity. During the 6 year follow-up period, 7 patients developed HCC. Maintaining ALT below 80 IU/l was found to prevent or delay HCC occurrence. In this study, we followed 55 HCV-infected individuals with fibrosis stage 3 (F3) for a period of 12 years, in order to assess which factors were important in HCC development, in these F3 patients when followed up for more than 10 years. Based on recent studies, the low ALT group was redefined as ALT < 30 IU/l. from <80 IU/l as in our previous paper. All patients were divided into low, moderate and high ALT groups.

## Patients and Methods

Fifty five CHC patients with F3 (male: 24; female: 31) who underwent initial IFN therapy were followed up from 1994 to 2005, at Kawasaki Hospital Okayama and Okayama Medical School Hospital, Okayama, Japan. The end-point of the study was the development of HCC. The total follow-up period was divided into 2 parts. The first half and second half each consisted of 6 years, respectively. The average observation period was 9.8 ± 2.3 years. The inclusion criteria for patients was; HCV RNA positive in sera; histologically diagnosed as CHC with F3, according to the classification of Knodell et al. and V. J Desmet (20–21); following initial IFN therapy, response was either complete biochemical response, (BR: ALT level < 30 IU/l); partial (PR: ALT between 30–80 IU/l); or non-response (NR: ALT > 80 IU/l), longer than 6 months post IFN. Patients who were sustained virological responders to IFN (HCV RNA negative in sera; ALT < 30 IU/l) over 6 months and had pre-existing HCC, were excluded. During the 12 year follow-up period, in the out-patient clinic patients underwent ultrasonography (US) every 6 months and on detection of liver cirrhosis (LC), every 3 months. Other imaging techniques such as computed tomography (CT), magnetic resonance imaging (MRI) were performed routinely once a year and when required. Serum ALT activity and other liver function tests were performed monthly. On suspicion of HCC, further imaging techniques, angiography and liver biopsy was carried out to confirm HCC diagnosis. The endpoint of the study was the development of HCC. Informed consent was obtained from all patients prior to this study. This study was performed under the approval guidelines of the ethical committee.

### Laboratory data analysis

The annual average serum ALT, PLT (platelet count) and other markers, from the beginning of the study till the end were calculated in all patients.

An annual average ALT (<30 IU/l) was considered low, while ALT (30–80) IU/l was taken as moderate, with ALT (>80 IU/l) being considered as high. In all patients, at least a minimum of 4–5 and a maximum of 12 ALT values/year were available, during the follow-up period. The decrease in PLT count over 12 years was calculated from the average PLT at the beginning of the study to the end of the study or on HCC development.

### Additional therapeutic procedures

- Anti-inflammatory drug therapy.
- Re-treatment with IFN or IFN and Ribavirin combination.
- Supplementary Vitamin E.
- All PR and NR patients underwent anti-inflammatory drug therapy such as; SNMC; 2–3 ampules (20 ml/ampule), 3 times/week and increased to a maximum of 4–5 ampules, 4–6 times a week}: UDCA; 300–600 mg/day to a maximum dose of 900 mg/day, with most patients receiving 600 mg/day and Shosaiko-to (7 g/day). Depending upon the ALT level, doses were readjusted accordingly. A total of 11 patients (BR = 2; PR = 5; NR = 4) patients were further re-treated with either IFN, PEG Intron (1.5 μg/kg once a week) or in combination with Ribavirin (600 ~ 1,000 mg/day). Unfortunately, all patients could not be re-treated with peginterferon and ribavirin due to costly reasons at the beginning of the study when all treatments were not covered by the health insurance and secondly due to side effects leading to patient reluctance. Supplementary Vitamin E therapy was given at a dose of 600 mg/day to a total of 13 patients.

### Statistical Analyses

The statistical analysis was carried out in two parts. At first, univariate analysis was performed using the statistical package SAS version 6.12. Next, to analyze the “time to event” clinical data, a Cox Regression Model was made. The following 5 covariates were entered into the model:

- The initial IFN response.
- The mean ALT over 3 years post initial IFN therapy.
- The presence of cirrhosis within 3 years post initial IFN therapy.
- Age and sex (known to affect the risk of HCC development) Results were expressed in mean ±S.D.

## Results

**Figure 1:**
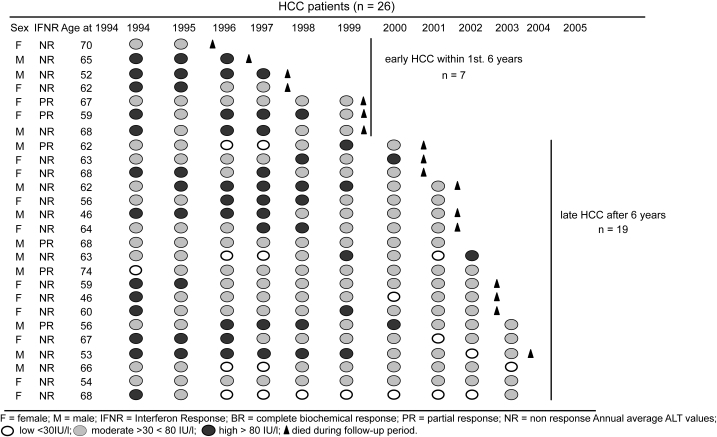
Change in annual average ALT during the follow-up period in HCC patients.

**Figure 2:**
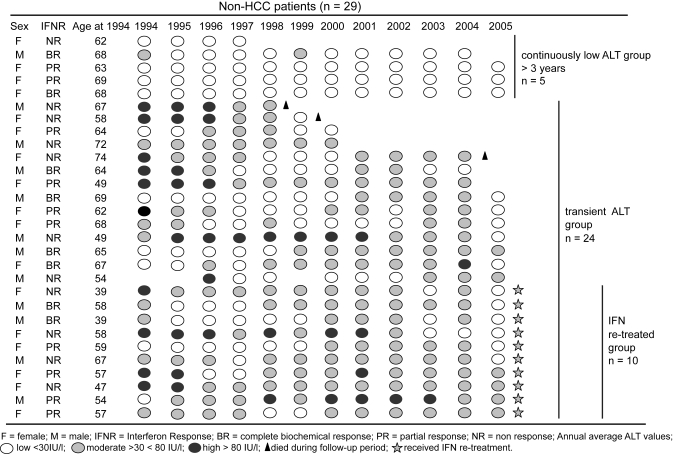
ALT pattern in the non-HCC group throughout the follow-up period.

The baseline characteristics of all 55 patients are given in Table 1. Over a span of 12 years (average 9.8 ± 2.3 years), 26 out of 55 patients developed HCC (NR = 20; PR = 6). No BR patient nor IFN retreated patient, except one, developed HCC. The annual incidence of HCC was 3.9%. All HCC and non-HCC patients are compared in Table 2. Univariate analysis to find the best predictor of HCC occurrence showed initial IFN response (*P* = 0.013), development of liver cirrhosis within a year of follow-up (*P* = 0.0006), and the 12 year annual average ALT activity (*P* = 0.0034), to be significantly related to HCC development. The Cox Proportional Hazard model, using 5 covariates, found the development of liver cirrhosis within 3 years of initial IFN therapy (Hazard Ratio: 3.39; 95% CI: 0.88–5.09; *P* = 0.05), and the 3 year annual average ALT activity, post initial IFN therapy (Hazard Ratio: 2.24; 95% CI: 1.07–4.71; *P* = 0.033), significantly related to HCC occurrence (Table 3). The IFN response, age and sex remained insignificant. When the 7 early HCC and 19 late HCC patients were compared (Table 4), development of liver cirrhosis within a year of follow-up (*P* = 0.0028) was the only significant variable associated with HCC development. The change in annual average ALT during the follow-up period in HCC patients in shown in Figure 1. Data is represented in order of early to late HCC from the top. While the early HCC group had no patient from the “L” ALT group (annual average ALT ≦ 30 IU/l), 8 (42%) patients in the late HCC group presented with low ALT one or more times during the follow-up period. Seventeen HCC patients died during the follow-up period of which, 14 (82.4%) were NR to initial IFN therapy. Figure 2 shows the annual average ALT pattern in the non-HCC group throughout the follow-up period. Five patients had continuously low ALT (<30 IU/l) for 3 years or more and did not receive IFN re-treatment. Within the transient ALT group (ALT 30–80 IU/l: n = 24), 10 patients (41.7%) received IFN re-treatment. Three patients in this group died before completion of study. Cause of death was suicide in one patient and liver failure in the other two.

## Discussion

Chronic HCV infection represents one of the major health problems throughout the world. Approximately 20%–30% of CHC patients will progress to LC, hepatic decompensation and HCC (22 ~ 24). In Japan, about 6%–8% HCV associated LC patients are assumed to be at a high risk for HCC development (25), that is to say, patients who are anti HCV positive and have reached the cirrhotic stage, have a greater possibility of developing HCC. Even in the non-cirrhotic group, the annual incidence of HCC increased from 0.07% in F0 and F1 to 2.2% in F3 patients with non-sustained virologic response to initial IFN therapy (5). Thus the F3 group of CHC patients are a vulnerable group who need to be monitored closely. High risk factors need to be demarcated in this group, in order to better understand how to prevent or delay HCC development. Some studies have shown that persistently high ALT is a major risk factor for HCC (26). Normalization of ALT with IFN (even without HCV eradication) has led to a less likelihood of HCC occurrence (27). In our previous study (18) we reported CHC patients with F3 who have a continuous elevation of ALT (>80 IU/l) for 2 years or more are at a high risk of developing HCC. Suppressing ALT activity below 80 IU/l by initial IFN followed by anti-inflammatory drugs, can prevent or delay HCC occurrence. In this study we aimed to find out whether even after a prolonged follow-up period (12 years), the ALT activity was still important in HCC development, as we have seen in an earlier study. In accordance with a study conducted by Okanoue et al. (28), the lower limit of ALT was adjusted to ≦30 IU/l and similar outcomes were observed in terms of HCC development. The continuously low ALT group (n = 5) did not develop HCC after 12 years, even in elderly patients, who did not receive IFN re-treatment. Though the number of patients with continuously low ALT (≦30 IU/l ) are few in this study, suppressing ALT ≦ 30 IU/l with anti-inflammatory drugs post IFN, may partly contribute in delaying HCC occurrence in patients, who are NR to initial IFN therapy. When HCC and non HCC patients were compared, none of the BR patients developed HCC. The initial IFN response, LC stage at beginning of study and the 12 year annual average ALT were significantly related to HCC occurrence. Analyzing the data with the use of the Cox Proportional Hazard model, LC within 3 years of initial IFN therapy and the 3 year annual average ALT post initial IFN, were found to be significantly related to HCC occurrence. Though in previous studies, populations at high risk of developing HCC in HCV infected patients include those of older age, male sex, non-response to initial IFN (25), in the present study, the average age of patients in both the HCC and non-HCC group was almost same (61.5 ± 7.1: 60.2 ± 9.1) years and there was no male predominancy in either group. However, HCC developed in 19 patients (almost 3 times the number) in the latter 6 years compared to 7 HCC patients in the first 6 years. Perhaps, advancement in age may have accelerated disease progression to HCC. In one of our previous studies (29) we found that oxidative stress markers such as reactive oxygen molecules (ROM) were significantly higher in HCV patients than controls and increased with age (P < 0.05). Since the non-HCC patients were also elderly, some other host factor in addition to oxidative damage, must be delaying HCC in this group. Studies (30) have shown that IFN re-treatment aids in re-achieving biochemical response in CHC patients. In 10 out of 11 patients in this study who were re-treated with IFN, IFN re-treatment might have helped to maintain a low ALT status or prevented further elevation of ALT, but a bigger patient population of IFN re-treated patients are required to establish this hypothesis. Efficacy of IFN re-treatment in BR, PR and even NR patients have been documented (31–32). As for the patient who developed HCC, irrespective of IFN re-treatment, it may be assumed that since the patient developed HCC within 18 months of IFN treatment, perhaps there was a pre-existing HCC, prior to IFN therapy for which HCC could not be prevented even with additional IFN. In the remaining 14 non-HCC patients, judging by their annual average ALT pattern, maintaining ALT below ≦80 IU/l on most occasions seemed to have contributed to HCC prevention until now. It remains to be seen how this non-HCC group progresses within the next 5 years. Probably the rate of progression to HCC will increase further. Continuously low ALT, biochemical or partial response to initial IFN therapy and IFN re-treatment may have prevented HCC, in these patients (33~35). From this study, it is apparent that long suppression of ALT below (<30 IU/l) may assist in lowering or delaying HCC occurrence in CHC patients with F3. Additional re-treatment with IFN, anti-inflammatory drugs and to some extent supplementary Vitamin E treatment (36) could have aided in lowering ALT. Though not significant, it was found that of the 13 patients who received supplementary Vitamin E treatment, only 2 developed HCC. Thus, keeping ALT activity continuously low (≦30 IU/l ), demarcating the moderately at risk ALT group (30–50 IU/L) and initiating IFN re-treatment and other supplementary treatments, even in elderly, non-responder patients, may be helpful in prevention or delay of HCC occurrence. However, small patient population and fewer number of IFN re-treated patients makes it difficult to decide the actual efficacy of IFN re-treatment in contributing to low ALT values, in this study. A bigger population of both IFN re-treated patients and continuously low ALT group needs studied further studies.

## Figures and Tables

**Table 1:** Patient Characteristics at baseline.

	n = 55
Age in years	60.8 ± 8.2
Sex (M/F)	(24/31)
H/O B.T (+)	43.60%
H/O L.D (+)	12.70%
H/O Alcohol (+)	7%
IFN response: BR/PR/NR	(8/16/31)
Genotype 1b	76%
HCV RNA > 1Meq/ml	61.00%
ALT(IU/l) (L < 30 INU; M 30–80 INU/l; H > 80 INU)	11/20/24
PLT (× 104 μl)	12.3 ± 3.6

**Abbreviations:** M/F: male/female; H/O: history of; BT: blood transfusion; LD: liver disease; IFN: interferon; BR: biochemical responder; PR: partial rresponder; NR: non-responder; ALT: alanine aminotransferase; L: low; M: moderate; H: high; PLT: platelet; Data are mean ± SD.

**Table 2:** Comparison of the clinical profile of HCC and non-HCC patients on univariate analysis (n = 55).

	HCC (+) n = 26	HCC (−) n = 29	P value
Age (in years)	61.5 ± 7.1	60.2 ± 9.1	NS
Sex (M/F)	(12/14)	(12/17)	NS
H/O B.T (+)	53.80%	34.50%	NS
H/O L.D (+)	7.70%	17.20%	NS
H/O Alcohol (+)	5%	10.30%	NS
IFN response: BR/PR/NR	(0/6/20)	(8/10/11)	0.0131*
Genotype 1b/other	(20/6)	(20/9)	NS
HCV RNA > 1Meq/ml	69.20%	55.20%	NS
LC within ‘94	30.80%	6.90%	0.0006*
**Biochemical values**			
12 year average ALT (‘94–’05) (<30 INU; 30–80 INU/l; >80 INU)	(4/17/5)	(11/14/4)	0.0034*
Decrease in PLT in 12 years (‘94–’05)	0.34 ± 0.31	0.33 ± 0.44	NS

**Abbreviations:** M/F: male/female; H/O: history of; BT: blood transfusion; LD: liver disease; IFN: interferon; BR: biochemical responder; PR: partial responder; NR: non-responder; ALT: alanine aminotransferase; PLT: platelet; HCC: hepatocellular carcinoma.

* **Note:** statistically significant by logistic regression analysis; Data are mean ± SD.

**Table 3:** Cox regression analysis to determine risk factors of HCC occurrence (n = 55).

Covariate	Hazard Ratio	95% CI	P value
Age	1.02	0.47–1.01	0.468
Sex	1.01	0.47–2.20	0.976
Initial	1.89	0.84–4.30	0.125
IFN response			
**LC within 3 years of initial IFN therapy**	**3.39**	**0.88–5.09**	**0.05**
**3 year Avg. ALT following intial IFN therapy**	**2.24**	**1.07–4.71**	**0.033**

**Abbreviations:** CI: confidence interval; IFN: interferon; LC: liver corrhosis; ALT: alanine aminotransferase.

**Table 4:** Comparison of the clinical profile of early HCC and late-HCC patients with stage 3 fibrosis (n = 26).

	Early HCC n = 7	Late HCC n = 19	P value
Age at 94	63.3 ± 6.2	60.8 ± 7.5	NS
Sex (M/F)	(3/4)	(9/10)	NS
H/O B.T (+)	57.10%	52.60%	NS
H/O L.D (+)	5.20%	5.30%	NS
H/O Alcohol (+)	0%	5.30%	NS
IFN response: PR/NR	(2/5)	(4/15)	NS
Genotype 1b/other	(5/2)	(21/6)	NS
HCV RNA > 1Meq/ml	57.10%	73.70%	NS
LC within ‘94	85.70%	10.50%	0.0028*
**Biochemical values**			
Avg. ALT at end pt. HCC(L/M/H) (<30 INU/l; 30–80 INU/l; >80 INU/l)	(0/4/3)	(4/13/2)	NS
Decrease in PLT till HCC (‘94–’05)	0.24 ± 0.36	0.37 ± 0.29	NS

**Abbreviations:** HCC: hepatocellular carcinoma; M/F: male/female; H/O: history of; BT: blood transfusion; LD: liver disease; IFN: interferon; PR: partial responder; NR: non-responder; PLT: platelet; LC: liver corrhosis; ALT: alanine aminotransferase.

* **Note:** statistically significant; Data are mean ± SD.
